# An efficiency analysis of high-order combinations of gene–gene interactions using multifactor-dimensionality reduction

**DOI:** 10.1186/s12864-015-1717-8

**Published:** 2015-07-01

**Authors:** Cheng-Hong Yang, Yu-Da Lin, Cheng-San Yang, Li-Yeh Chuang

**Affiliations:** Department of Electronic Engineering, National Kaohsiung University of Applied Sciences, Kaohsiung, Taiwan; Department of Plastic Surgery, Chia-Yi Christian Hospital, Chiayi, Taiwan; Department of Chemical Engineering & Institute of Biotechnology and Chemical Engineering, I-Shou University, Kaohsiung, Taiwan

**Keywords:** SNPs, Gene–gene interactions, Multifactor dimensionality reduction

## Abstract

**Background:**

Multifactor dimensionality reduction (MDR) is widely used to analyze interactions of genes to determine the complex relationship between diseases and polymorphisms in humans. However, the astronomical number of high-order combinations makes MDR a highly time-consuming process which can be difficult to implement for multiple tests to identify more complex interactions between genes. This study proposes a new framework, named fast MDR (FMDR), which is a greedy search strategy based on the joint effect property.

**Results:**

Six models with different minor allele frequencies (MAFs) and different sample sizes were used to generate the six simulation data sets. A real data set was obtained from the mitochondrial D-loop of chronic dialysis patients. Comparison of results from the simulation data and real data sets showed that FMDR identified significant gene–gene interaction with less computational complexity than the MDR in high-order interaction analysis.

**Conclusion:**

FMDR improves the MDR difficulties associated with the computational loading of high-order SNPs and can be used to evaluate the relative effects of each individual SNP on disease susceptibility. FMDR is freely available at http://bioinfo.kmu.edu.tw/FMDR.rar.

**Electronic supplementary material:**

The online version of this article (doi:10.1186/s12864-015-1717-8) contains supplementary material, which is available to authorized users.

## Background

Large single nucleotide polymorphisms (SNPs) research projects across the human genome are important studies for biological and biomedical science, with many researchers seeking to use SNPs as predictors for susceptibility to disease. Traditional approaches to identify SNP interactions usually use bio-statistical methods such as contingency tables combined with *k*-fold cross-validation, but the vast number of possible combinations makes the application of traditional methods difficult. Therefore, current research is aimed at combining biostatistics and machine learning in family-based and case–control association studies [1–8].

Multifactor dimensionality reduction (MDR) [9] is a well-known hybrid technology that combines a 2-way contingency table, *k*-fold cross-validation, and a dimensionality reduction technique. MDR belongs to a group of non-parametric statistical methods used to determine high-order gene–gene interactions in case–control studies [9, 10]. Typically, multi-locus genotypes are classified into high-risk and low-risk classes, allowing the number of genotype predictors to be effectively reduced from *n* dimensions to one dimension. This reduction influences the contingency table allowing for the quick computation of statistics including the accuracy rate, odds ratio (*OR*), *P*-value, etc. Many modifications and extensions to MDR have been proposed and these can be divided into three groups. The first group contains modifications and combinations of biostatistics in MDR; this group includes entropy-based interpretation methods [11], the use of *OR* [12], generalized linear models [13], log-linear methods [14], Bayesian posterior probability [15], and model-based methods [16]. The second group focuses on particular data problems, such as imbalanced data [17, 18], permutation testing [19], and missing data [20]. These extensions and modifications of MDR have been used to address different situations encountered in disease analysis. Many disease studies have thus successfully employed MDR to detect interactions between particular genes, including those for coronary artery disease [21, 22], hypertension [23–25], bladder cancer [26], and autism [27]. Finally, the third group aims to reduce MDR computational time, using methods including parallel implementations [28] and the use of hardware graphics processing units (GPUs) [29, 30]. Although these studies use GPUs to reduce MDR running time, the problem of factorial operation in MDR still presents a challenge.

This study seeks to develop a new framework to improve MDR computational times in investigations of high-order gene–gene interaction. The framework retains the significant factors to reduce the number of multi-locus evaluations in MDR. Improvements in computational time were measured over 100 runs on a simulation data set and a genome-wide analysis of chronic dialysis epistasis.

## Method

### MDR algorithm

MDR is an attribute construction approach that reduces the data dimensionality by seeking to identify combinations of multi-locus genotypes that are associated with either high-risk or low-risk groups. The combination of two or more locus genotypes into a single attribute can be used to effectively estimate the risk associated with gene–gene interactions in relation to a disease. This study uses the imbalanced functions proposed by Yang *et al*. [17]. MDR can be divided into five separate processes. In the first step, the data are divided into 10 parts for ten-fold cross-validation. Nine-tenths of the data are classified as training sets and the remaining 1/10 is used for testing. The second step is the construction of a contingency table. For a given interaction order *n*, *n* SNPs are selected from the data set. *L* is defined as a set of multi-locus genotypes at *n* loci and/or environmental factors. *L* can be represented as an *n*-dimensional vector:

1 $$ L = \left\{{l}_1,{l}_2,{l}_3, \dots, {l}_n\right\} $$

where *l* represents an SNP factor and/or environmental factor.

Next, *L* is used to calculate the case–control ratios for each multi-locus genotype. The ratio between cases and controls is evaluated by Equation (2).

2 $$ f(L)=\frac{N^{*}\left[{\displaystyle {\sum}_{j=1}^{P^{*}}u\left(L,{P}_j\right)}\right]}{P^{*}\Big[{\displaystyle {\sum}_{j=1}^{N^{*}}u\left(L,{N}_j\right)\Big]}} $$

where

$$ u\left(L,A\right)=\left\{\begin{array}{cc}\hfill 1\hfill & \hfill \forall l\in A\hfill \\ {}\hfill 0\hfill & \hfill \forall l\notin A\hfill \end{array}\right.\kern1.5em ,\forall l\in L $$

where the cases are labelled *P* and the controls are labelled N. P^*^ and N^*^ respectively represent the sizes of cases and controls in the training set. Here *j* represents the index of samples in the cases and controls. *P*_*j*_ represents the *j*^th^ sample among the cases and *N*_*j*_ represents the *j*^th^ sample among the controls. *u*(*L*, *P*_*j*_) represents a match (given a score of “1”) if all multi-locus genotypes *l* in vector *L* match *P*; a mismatch is given a score of “0”. *u*(*L*, *N*_*j*_) represents a match (given a score of “1”) if all multi-locus genotypes *l* in vector *L* match *N*; a mismatch is given a score of “0”. For example, a 2-order interaction model consisting of SNP1 and SNP2 has nine multi-locus genotypes, i.e., AA-AA, AA-Aa, AA-aa, Aa-AA, Aa-Aa, Aa-aa, aa-AA, aa-Aa and aa-aa. The AA represents the homozygous reference genotype, while Aa represents the heterozygous genotype and aa represents the homozygous variant genotype. In the first multi-locus genotype (AA-AA), the ${\displaystyle {\sum}_{j=1}^{P^{*}}u\left(L,{P}_j\right)}$ includes 88 samples matching AA-AA among the cases and ${\displaystyle {\sum}_{j=1}^{N^{*}}u\left(L,{N}_j\right)}$ includes 90 samples matching AA-AA among the controls. Evaluation with Eq. 2 yields a value of 0.978, which is computed by (88 × 300)/(90 × 300); P^*^ and N^*^ are respectively 300 samples among both the cases and controls.

After the ratio calculation, each *L* is labelled 'H' ("high") if the ratio of cases to controls is equal to or greater than a threshold of *T* (=1); otherwise it is labelled 'L' ("low"). Once all *L*s are labelled ‘H’ or ‘L’, a new binary attribute is created by pooling the high-risk genotype combinations into one group and the low-risk genotype combinations into another group. This means that the four frequencies (TP, FP, TN, and FN) can be computed in a 2-way contingency table. Finally, each possible *L* computes a training classification error rate for each *n*-way interaction in the training set. The classification error rate is given by Equation (3).

3 $$ \mathrm{Classification}\ \mathrm{error}\ \mathrm{rate}=0.5\times \left(\frac{FN}{TP+FN}+\frac{FP}{FP+TN}\right) $$

Among all *n* SNP combinations, the best model with the minimum classification error rate is selected by the training step. The third step evaluates the remaining 1/10 of the original data set (i.e., the independent test data). This step creates an MDR attribute for the testing set using the *n* SNPs that have the minimum training classification error rate. In addition, the best model in each cross-validation is collected and named the cross-validation consistency (CVC). In the fourth step, the procedure is repeated 10 times (i.e., ten-fold cross-validation) so that each sample is included in the testing set once, and the resulting classification error rates of each of the ten models in CVC are averaged. In the last step, the best MDR model with the highest frequency in CVC is selected.

### Fast MDR algorithm (FMDR)

**Fig. 1:**
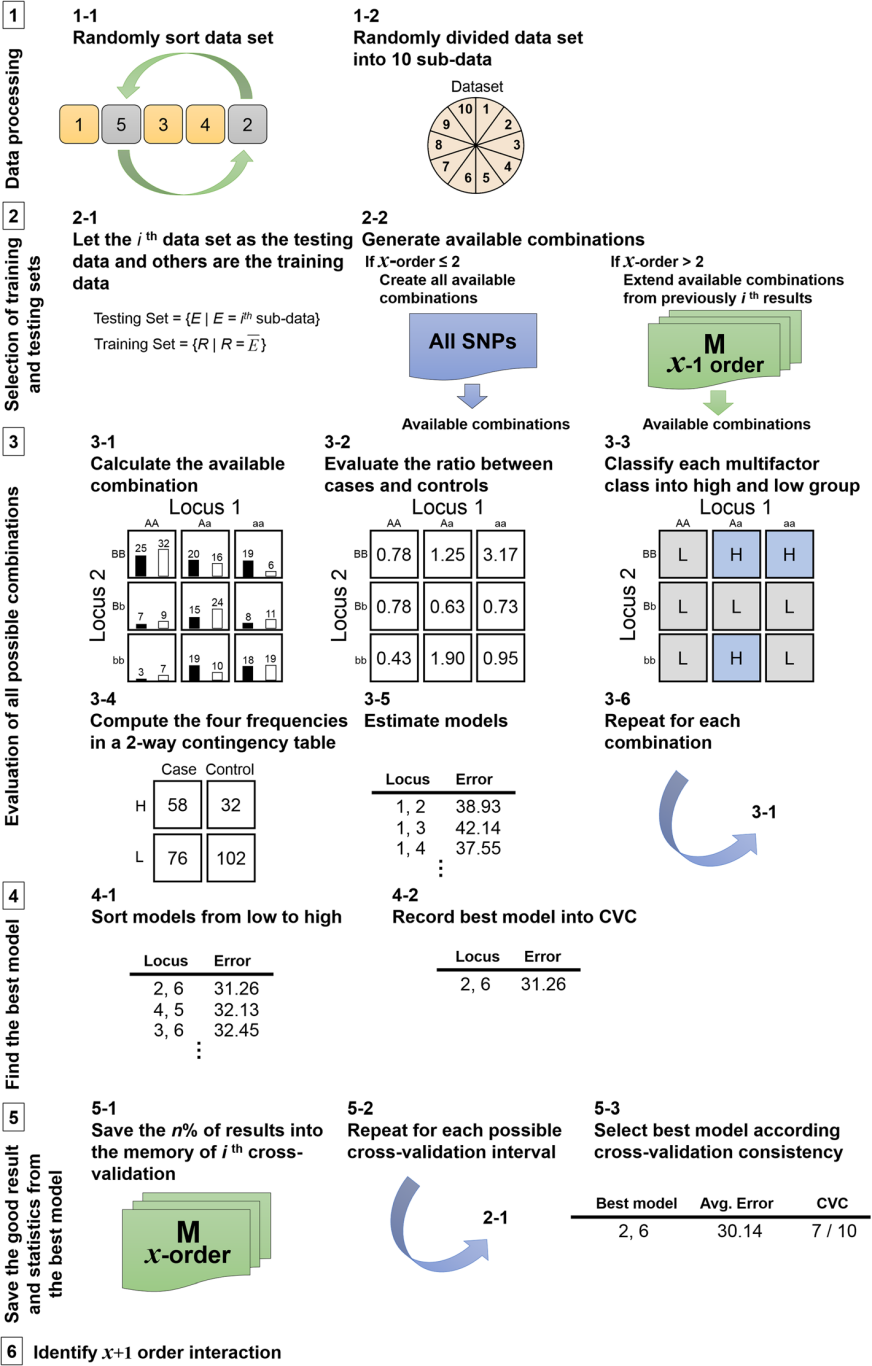
FMDR flowchart

FMDR proposes a new framework to improve the MDR computational time. Figure 1 shows the FMDR flowchart consisting of five steps: (1) data processing, (2) selection of training and testing sets, (3) evaluation of all possible combinations, (4) identifying the best model, and (5) statistical analysis of the best model. In the FMDR, the number of selected SNPs is limited to two at the outset. The framework is represented by the thick frame in steps (2) and (5) (Fig. 1). In step (2), the framework checks whether or not the number of loci is equal to two. If yes, all available two-order locus combinations amongst the loci are created and regarded as conditions. All these conditions are then used to evaluate the contingency table (step (3)), and the classification error rate in each combination is estimated by Equation (3) (step (4)). In step (5), all two-order locus combinations are sorted based on the classification error rate, and then the results of the best *n*% combinations with the minimum classification error rate are saved into the *i*^th^ memory where *i* is the *i*^th^-fold cross-validation. When ten cross-validations are computed, the best 2-loci model is output to show related gene–gene interaction information. If the number of order exceeds two (i.e., *m*-loci, *m* > 2), each cross-validation uses the corresponding memory and the recorded results of the best *n*% combinations to create the available combinations (go to step (2)), i.e., conditions. In step (3), these conditions are evaluated using the contingency table, and the classification error rates of the conditions are estimated in step (4). The results are then sorted and the best *n*% combinations are saved into *i*^th^ memory to analyze the next interaction order. This process tremendously reduces the number of available combinations. The processes are repeatedly implemented until the defined number of selected SNPs is analyzed.

### Illustrative example to FMDR and statistical analysis

The supplementary Additional file 1 provides an example to illustrate how the FMDR works, and the supplementary Additional file 2 explains the statistical analysis method.

## Results

### Results on the simulated data set

**Fig. 2:**
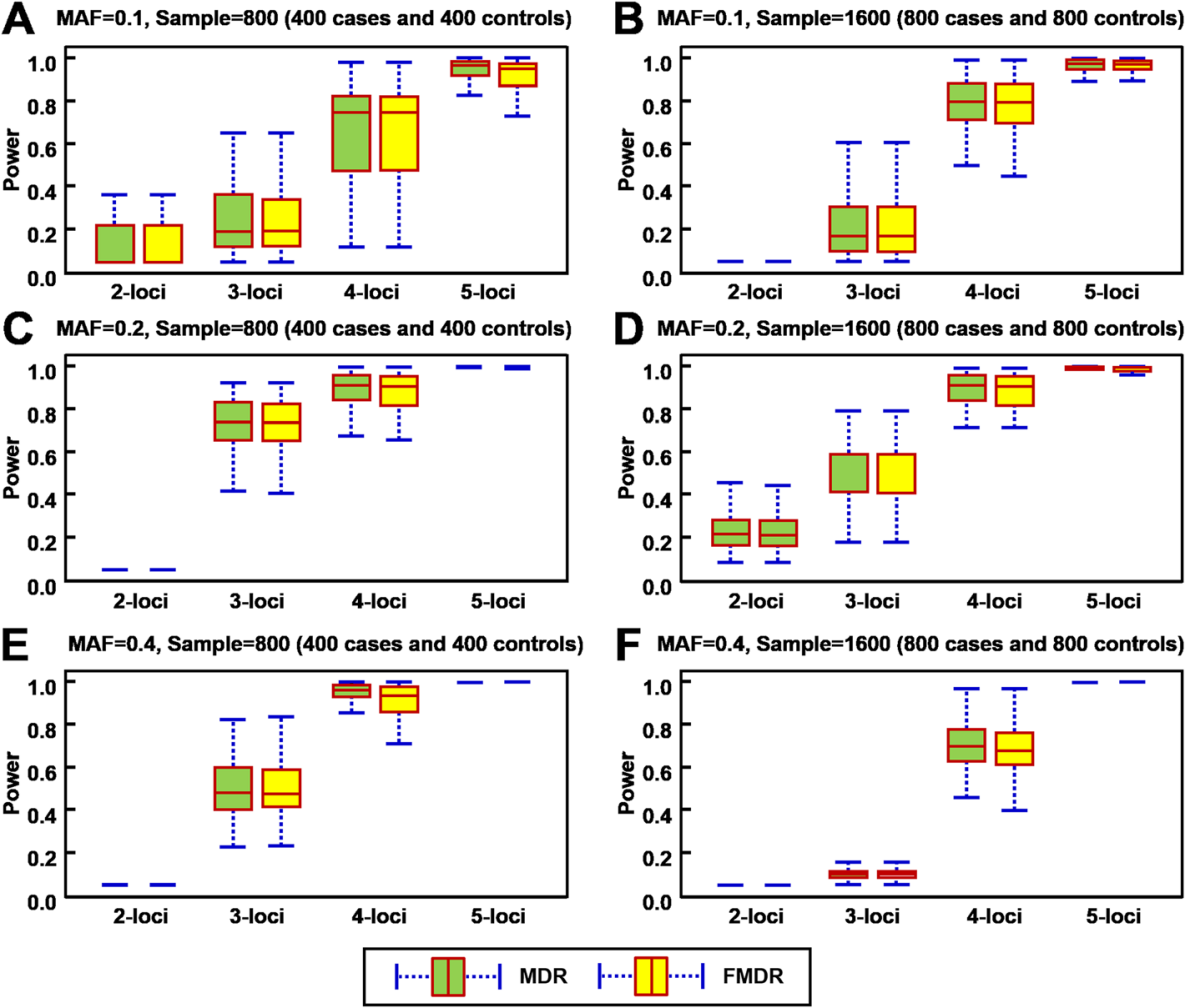
Performance comparison between MDR and FMDR on six simulated models for different minor allele frequencies (MAFs) and different sample sizes (**a**–**f** of Fig. 2). For all models, heritability *h* ^2^ = 0.2, and MAF includes 0.1, 0.2, and 0.4. For each model, 100 datasets are generated by randomly sorted samples. The figures show the box plot, where the boundary of the box closest to zero indicates the 25^th^ percentile, a line within the box marks the median, and the boundary of the box farthest from zero indicates the 75^th^ percentile. Error bars near the top and bottom of the boxes respectively indicate the 90^th^ and 10^th^ percentiles

All simulated models set the 50 attributes with a heritability of 0.2. The minor allele frequencies (MAFs) were 0.1, 0.2, and 0.4. The sample sizes were 800 and 1600, in which the total number of cases is equal to the total number of controls. The simulation data was generated using GAMETES, software used for generating complex *n*-loci models with random architectures [31]. The settings and results of the six models are shown in Table 1. Figure 2 shows the power analysis box plots of six models. A summary of the six simulation data set shows that the difference between MDR and FMDR was statistically significant for 4-loci and 5-loci, but there was only a slight difference between the averages of the two methods.

**Table 1:** A paired *t*-test comparison of the power analysis between MDR and FMDR for 2- to 5-loci

Model^1^	2-loci			3-loci			4-loci			5-loci		
	MDR	FMDR	*P*-value^2^	MDR	FMDR	*P*-value	MDR	FMDR	*P*-value	MDR	FMDR	*P*-value
	Mean (SD)	Mean (SD)		Mean (SD)	Mean (SD)		Mean (SD)	Mean (SD)		Mean (SD)	Mean (SD)	
Model 1	0.12 (±0.11)	0.12 (±0.11)	−^3^	0.27 (±0.21)	0.27 (±0.21)	−	0.67 (±0.21)	0.67 (±0.21)	0.17	0.94 (±0.08)	0.91 (±0.11)	<0.001
Model 2	0.05 (±0.00)	0.05 (±0.00)	−	0.24 (±0.18)	0.24 (±0.18)	−	0.78 (±0.14)	0.78 (±0.14)	0.65	0.96 (±0.04)	0.96 (±0.05)	0.001
Model 3	0.05 (±0.00)	0.05 (±0.00)	−	0.73 (±0.14)	0.72 (±0.14)	0.01	0.90 (±0.08)	0.88 (±0.09)	0.003	0.99 (±0.01)	0.99 (±0.01)	<0.001
Model 4	0.24 (±0.10)	0.24 (±0.10)	−	0.50 (±0.14)	0.50 (±0.14)	0.32	0.88 (±0.09)	0.87 (±0.09)	0.01	0.99 (±0.02)	0.98 (±0.03)	0.001
Model 5	0.06 (±0.01)	0.06 (±0.01)	−	0.50 (±0.13)	0.50 (±0.12)	0.89	0.95 (±0.06)	0.91 (±0.10)	<0.001	1.00 (±0.00)	1.00 (±0.00)	−
Model 6	0.05 (±0.00)	0.05 (±0.00)	−	0.11 (±0.04)	0.11 (±0.04)	−	0.70 (±0.12)	0.70 (±0.12)	0.001	1.00 (±0.00)	1.00 (±0.00)	0.001

^1^Model 1: MAF = 0.1, sample = 800 (400 cases and 400 controls), Model 2: MAF = 0.1, sample = 1600 (800 cases and 800 controls), Model 3: MAF = 0.2, sample = 800 (400 cases and 400 controls), Model 4: MAF = 0.2, sample = 1600 (800 cases and 800 controls), Model 5: MAF = 0.4, sample = 800 (400 cases and 400 controls), Model 6: MAF = 0.4, sample = 1600 (800 cases and 800 controls); ^2^ *P*-value were estimated from pairwise *t*-test; ^3^-: the same power analyses between MDR and FMDR

**Fig. 3:**
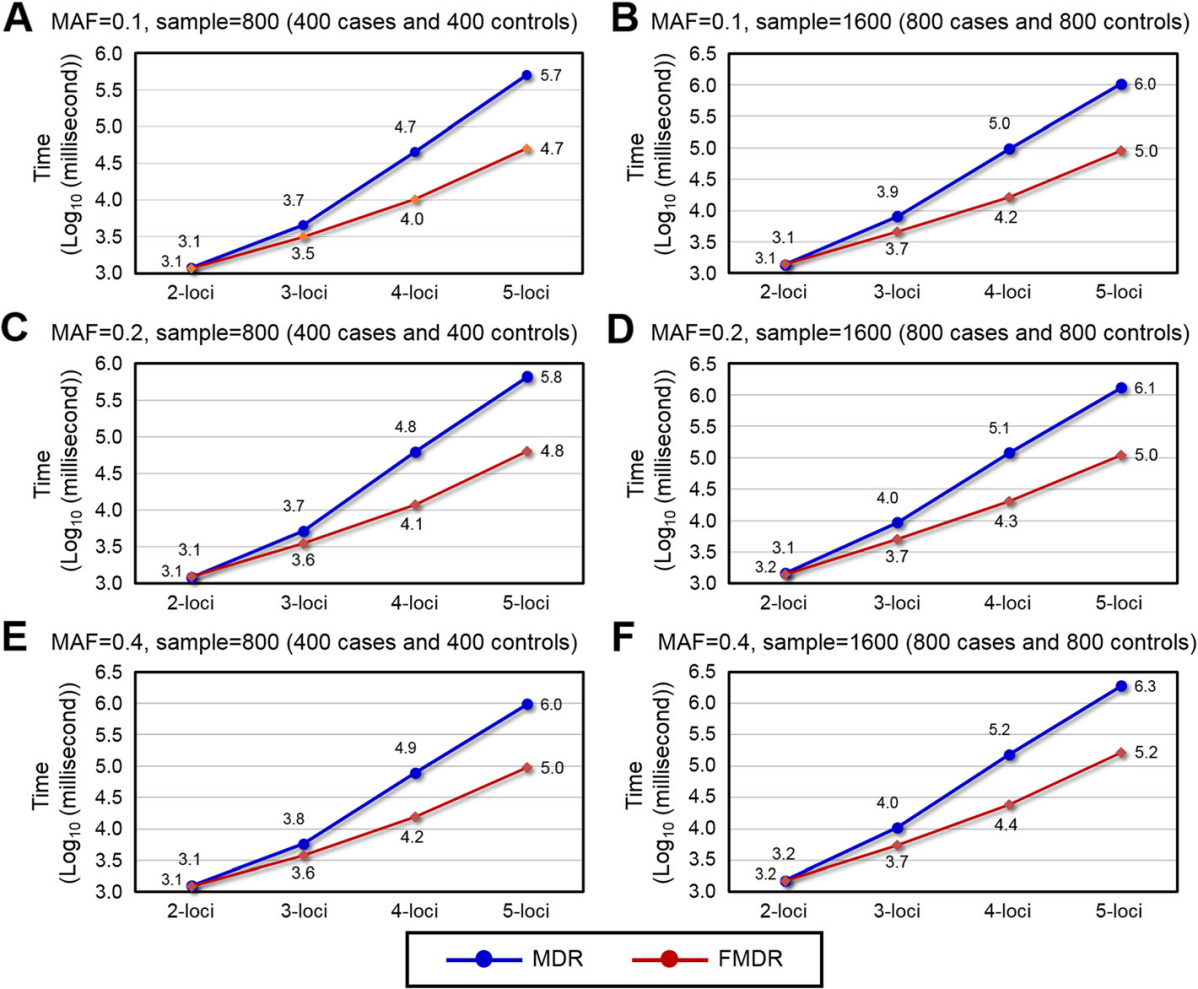
MDR and FMDR execution times on six simulated models for different MAFs and different sample sizes (a-f of Fig. 3). The horizontal axis represents the execution time in log_10_ milliseconds, while the vertical axis represents the number of loci in the model

Figure 3 showed the execution times for the simulation data sets. The MDR execution times in all locus orders were collected in a stand-alone test. The total of all FMDR execution times for all locus orders was collected since FMDR uses a continual analysis strategy. For the six simulation data sets, MDR and FMDR required similar durations to implement the 2-loci analysis. When comparing 2-loci and *n*-loci (*n* = 3, 4, 5) in model 1, the growth times between MDR and FMDR for 3-loci to 5-loci were 3.796 *vs*. 2.691, 38.279 *vs*. 8.712, and 424.18 *vs*. 43.260 (milliseconds). Similarly, Figure C1 of supplementary Additional file 3 compares the 2-loci and *n*-loci in models 3–6. We compared the growth time between 800 and 1600 samples in different MAFs. For MAF = 0.1, MDR and FMDR for 2-loci to 5-loci were 1.162 *vs*. 1.197, 1.796 *vs*. 1.452, 2.140 *vs*. 1.599, and 2.063 *vs*. 1.774. Similarly, Figure D1 of supplementary Additional file 4 shows the growth times between MDR and FMDR in other MAFs (i.e., MAF = 0.2 and MAF = 0.4). The results for the simulation data sets showed that FMDR effectively reduces MDR computational time.

### Results on the chronic dialysis data set

The 77 mitochondrial SNPs in the D-loop region of chronic dialysis patients were obtained from investigations conducted by Chen *et al*. [32] that enrolled 193 chronic dialysis patients and 704 healthy controls from unrelated ethnic Chinese in Taiwan. The results revealed that chronic peritoneal dialysis patients suffer from higher oxidative stress than healthy subjects; this elevated oxidative stress alters the number of copies of mtDNA in peripheral leukocytes. The possible complicated networks with direct or indirect cross-communication among the 77 SNP candidates were explained. The ratio of controls (*n* = 704) to cases (*n* = 193) was 3.65:1. We randomly sorted the samples in the data set to generate 100 data sets each of which was then divided into ten groups for ten-fold cross-validation. The ratios of cases to controls amongst 1000 training sets range from 3.41–3.95, with a mean (SD) ratio of 3.65 (0.10). Each data set was used once to test MDR and FMDR.

**Fig. 4:**
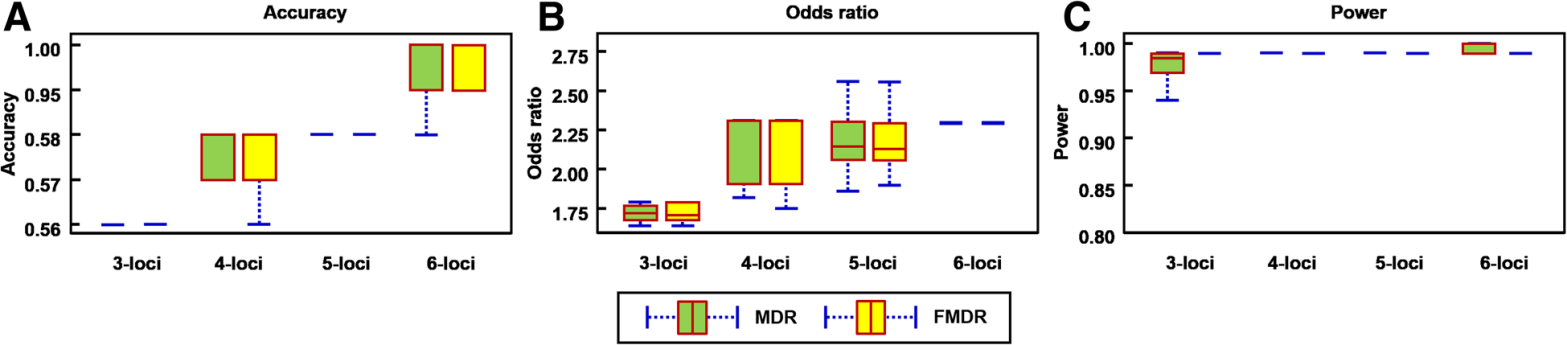
Performance comparison between MDR and FMDR for the chronic dialysis data set. (**a**) accuracy box plot for MDR and FMDR, (**b**) *OR* box plot for MDR and FMDR, and (**c**) power analysis box plot for MDR and FMDR for 100 tests. For each test, the samples in the data set are randomly sorted, and then applied to MDR and FMDR

For the 100 tests, we summed up the frequencies of the results based on the cross-validation consistency (CVC) and the classification error rate in each test. The accuracy and *OR* of the best candidate model was evaluated. Table 2 shows the best, worst, and mean (±SD) in the 100 tests for MDR and FMDR. For the 3- –6-loci models producing the best accuracy amongst the 100 tests, both MDR and FMDR had the same candidate model, and also had the same accuracy and *OR*. In the models for 3- to 6-loci with the lowest accuracy amongst the 100 tests, MDR and FMDR were different slightly, and the accuracy and *OR* also differed. A box plot was used to compare the two methods for 3-, 4-, 5-, and 6-loci interactions. Figure 4a and b respectively shows the accuracy and *OR* box plot of MDR and FMDR. Paired *t*-test comparison results indicate that the accuracy and *OR* values for 3- –6-loci analysis over 100 test runs were similar for both MDR and FMDR. Figure 4c shows the box plot of the power results of MDR and FMDR for four order interactions. As the order of interaction increases, both MDR and FMDR shows increasing power values. All powers of MDR and FMDR exceeded 0.8. A summary of the 100 test runs shows that the difference between MDR and FMDR was statistically significant for 3- and 6-loci, but the average difference between the two methods is very slight, i.e., −0.011 at 3-loci and 0.002 at 6-loci. In addition, the powers in the 4- and 5-loci analysis over 100 test runs are similar for both MDR and FMDR.

**Table 2:** Analysis results of the chronic dialysis data sets from MDR and FMDR

Models	Method	3-loci		4-loci		5-loci		6-loci	
		MDR	FMDR	MDR	FMDR	MDR	FMDR	MDR	FMDR
Best									
Candidate model		40,56,64	40,56,64	21,59,64,71	21,59,64,71	21,59,62, 64,71	21,59,62, 64,71	21,45,59, 62,64,71	21,45,59, 62,64,71
Consistency		2 / 10	2 / 10	4 / 10	2 / 10	1 / 10	1 / 10	2 / 10	3 / 10
Accuracy		0.56	0.56	0.58	0.58	0.58	0.58	0.60	0.60
*OR* (95 % CI)		1.79	1.79	1.91	1.91	2.13	2.13	2.30	2.30
		(1.27–2.52)	(1.27–2.52)	(1.38–2.63)	(1.38–2.63)	(1.54–2.94)	(1.54–2.94)	(1.66–3.18)	(1.66–3.18)
Worst									
candidate model		45,56,62	45,56,65	40,45,56,62	19,21,34, 56	41,43,45, 56,62	40,56,64, 71,77	4,21,56, 60,62,70	40,21,56, 64,71,77
Consistency		1 / 10	1 / 10	2/10	1/10	1 / 10	1 / 10	1 / 10	1 / 10
Accuracy		0.56	0.56	0.57	0.56	0.57	0.57	0.58	0.59
*OR* (95 % CI)		1.64	1.65	1.82	1.78	1.86	1.90	2.13	2.28
		(1.18–2.27)	(1.19–2.31)	(1.31–2.54)	(1.27–2.49)	(1.34–2.60)	(1.36–2.64)	(1.50–3.03)	(1.59–3.26)
Accuracy									
Mean (SD)		0.56 (±0.00)	0.56 (±0.00)	0.58 (±0.01)	0.58 (±0.01)	0.58 (±0.002)	0.58 (±0.002)	0.60 (±0.01)	0.60 (±0.01)
*P*-value^1^		−^2^		0.07		0.32		0.85	
*OR*									
Mean (SD)		1.73 (±0.05)	1.73 (±0.05)	2.04 (±0.19)	2.03 (±0.19)	2.21 (±0.18)	2.17 (±0.19)	2.31 (±0.08)	2.30 (±0.08)
*P*-value		0.66		0.44		0.03		0.46	
Power									
Mean (SD)		0.97 (±0.03)	0.99 (±0.02)	0.99 (±0.02)	0.99 (±0.02)	0.99 (±0.003)	0.99 (±0.002)	0.99 (±0.004)	0.99 (±0.003)
*P*-value		<0.001		0.83		0.71		<0.001	

^1^ *P*-value were estimated from pairwise *t*-test; ^2^-: the same accuracies between MDR and FMDR

**Fig. 5:**
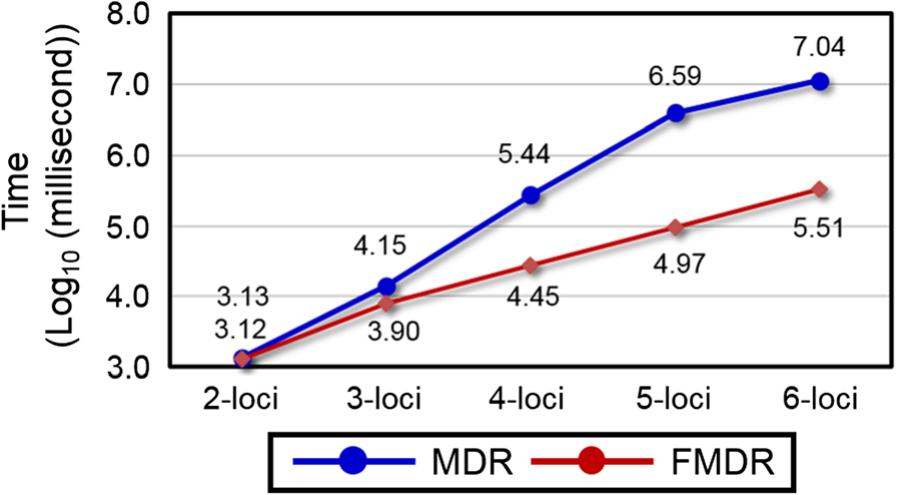
Execution times of MDR and FMDR for the real data set with chronic dialysis. The horizontal axis represents execution time in log_10_ milliseconds, while the vertical axis represents the number of loci in the model

In Fig. 5, for the real data set, both MDR and FMDR required similar amounts of time to implement the 2-loci analysis. When comparing 2-loci and *n*-loci (*n* = 3, 4, 5, 6), the growth times between MDR and FMDR are 10.40 *vs*. 5.95, 200.16 *vs*. 21.08, 2880.95 *vs*. 70.93, and 8081.88 *vs*. 245.81. These results ind-+icate that the proposed framework can reduce the execution times required by MDR for high-order interaction analysis.

## Discussion

The substantial computational limits of MDR make it difficult to detect nonlinear interactions of high-order combinations of SNPs amongst a large number of SNPs. Determining all combinations of SNPs in MDR entails calculating *C*(*N*,*M*) × *V* = *N*!/[*M*!(*N*-*M*)!] × *V* combinations, where *N* is the total number of SNPs, *M* is the number of factors considered for a model, and *V* is the number of cross-validation intervals. In big-O notation, MDR has a time complexity of O(n!).

Exhaustive search approaches, e.g., genetic algorithm (GA) [10] and ant colony optimization (ACO) [33] are important for improving MDR computational times. GA and ACO use small combinations to find the acceptable *n*-loci gene–gene interaction model in a huge combination space, thus effectively reducing the computational time requirements. However, all parameters can influence the results of detected gene–gene interaction. The parameters of population size, generation size, random seed, and algorithm setting (e.g., mutation probability in GA and pheromone in ACO) are difficult to define to successfully find the *n*-loci gene–gene interaction model for data sets of different sizes, i.e., sample size and SNP size. Therefore, current research directions focus on the use of software and hardware to improve MDR computational times.

Many researchers employ software [28] and hardware [29, 30] techniques to speed up MDR. Bush *et al*. proposed a framework which divides the MDR processes into three classes: (1) a data handling class, (2) a model generation and processing class, and (3) a result storage class. These three modular classes were implemented in parallel, finding that parallel MDR can be used to analyze high-order interactions of small data sets and can feasibly perform lower-end genome-wide analyses. Greene *et al*. and Sinnott-Armstrong *et al*. [29, 30] employed modern computer Graphics Processing Units (GPUs) to speed up MDR since GPUs have a higher memory bandwidth and computational capability than Central Processing Units (CPUs). Still, the factorial increase of time complexity remains an obstacle.

The FMDR procedure is a type of greedy search strategy [34], and is based on joint effect property [35]. The joint effect can be divided into the three effects: (1) overall effect, (2) *n*-order interaction effect, and (3) main effect. In epistasis, overall effect indicates the common effect amongst *n* risk factors. The main effect indicates any effect(s) could serve as a guide to determining the correct multi-locus interaction. The *n*-order interaction effect indicates the least proper subset of the loci also interacts epistatically. The highly-associated SNPs have a high probability of being a significant factor in the next-order interaction. A low classification error rate in an MDR model indicates a high statistically significant risk of *n*-loci effects. Suppose all 2-loci combinations in four SNPs are sorted according the classification error rate as {SNP_a_, SNP_b_}, {SNP_b_, SNP_c_}, {SNP_a_, SNP_c_}, {SNP_a_, SNP_d_}, …, {SNP_b_, SNP_d_}. The {SNP_a_, SNP_b_} is the best model in 2-loci gene–gene interaction. The {SNP_b_, SNP_c_} and {SNP_a_, SNP_c_} combinations are both probably significant models for gene–gene interaction, but neither is the best model. SNP_c_ has the highest probability of joining the 3-loci gene–gene interaction because it’s strong association with SNP_a_ and SNP_b_ (i.e., 2-order interaction effect). On the other hand, {SNP_b_, SNP_d_} is the worst model; it means that adding SNP_d_ via SNP_b_ into the gene–gene interaction network is the least likely scenario. SNP_d_ has a high probability of being added via the SNP_a_ effect because {SNP_a_, SNP_d_} belongs to the top model with a low classification error rate. Therefore, the {SNP_b_, SNP_d_} can be deleted, and all combinations based on {SNP_b_, SNP_d_} in 3-loci combination are not evaluated. These properties allow us to apply the greedy search strategy to find the significant gene–gene interaction model. Moreover, FMDR only sets one parameter to select the number of best combinations with the low classification error rate, which are then saved into the memory. We suggest the optimal choice for *n* is the dynamic adjustment according to the order of interaction, i.e., *n* = 2 with 2-order gene-gene interaction and *n* = 3 with 3-order gene-gene interaction.

The idea behind FMDR is the retention of good results for high-order interaction, indicating the available combinations are generated from *n*% good results, i.e., *n*% results × *N* combinations, where *N* is the total number of SNPs. Therefore, FMDR has a time complexity of O(n). The execution time of FMDR is much shorter than that of MDR in high-order gene–gene interactions because FMDR effectively decreases the number of possible unnecessary computations. FMDR includes the following advantages: (1) FMDR can effectively reduce the computational time required by MDR for high-order interactions, (2) the best model has a low classification error rate and a high sensitivity for disease prediction, and (3) FMDR can easily be combined with existing MDR methods.

## Conclusions

FMDR based on the joint effect property reduces MDR computational time by retaining results for higher interactions. The retained number of results can be formularized and improved using statistical methods and mathematic theories in future work. The time complexity can be easily computed by estimation of a function. We suggest that the function be designed as a dynamic adjustment based on the data set size and the order of interaction. The flexible framework underlying FMDR can effectively improve the limitations of existing MDR methods in finding high-order interactions.

## Additional files

Additional file 1:
**Example to illustrate the FMDR calculation process.**
Additional file 2:
**Statistical analysis.**
Additional file 3:
**Figure to illustrate the growth times for comparing 2-loci and** ***n*** **-loci.**
Additional file 4:
**The figure for Growth times comparing 800 and 1600 samples in three MAFs.**
